# Nature of bacterial colonization influences transcription of mucin genes in mice during the first week of life

**DOI:** 10.1186/1756-0500-5-402

**Published:** 2012-08-02

**Authors:** Anders Bergström, Matilde B Kristensen, Martin I Bahl, Stine B Metzdorff, Lisbeth N Fink, Hanne Frøkiær, Tine R Licht

**Affiliations:** 1 Gut Ecology Group, Department of Food Microbiology, National Food Institute, Technical University of Denmark, Mørkhøj Bygade 19, Søborg, 2860, Denmark; 2 Department of Basic Sciences and Environment, Faculty of Life Sciences, University of Copenhagen, Copenhagen, Denmark; 3 Center for Biological Sequence Analysis, Department of Systems Biology, Technical University of Denmark, Lyngby, Denmark

**Keywords:** Germ free mice, Monocolonized, qPCR, LinRegPCR, Postnatal transcription onset, Probiotics, *Lactobacillus acidophilus* NCFM, *Escherichia coli* Nissle, 16S rRNA

## Abstract

**Background:**

Postnatal regulation of the small intestinal mucus layer is potentially important in the development of adult gut functionality. We hypothesized that the nature of bacterial colonization affects mucus gene regulation in early life.

We thus analyzed the influence of the presence of a conventional microbiota as well as two selected monocolonizing bacterial strains on the transcription of murine genes involved in mucus layer development during the first week of life.

Mouse pups (N = 8/group) from differently colonized dams: Germ-free (GF), conventional specific pathogen free (SPF), monocolonized with either *Lactobacillus acidophilus* NCFM (*Lb*) or *Escherichia coli* Nissle (*Ec*) were analyzed by qPCR on isolated ileal tissue sections from postnatal days 1 and 6 (PND1, PND6) after birth with respect to: (i) transcription of specific genes involved in mucus production (*Muc1*-*4*, *Tff3*) and (ii) amounts of 16S rRNA of *Lactobacillus* and *E. coli*. Quantification of 16S rRNA genes was performed to obtain a measure for amounts of colonized bacteria.

**Results:**

We found a microbiota-independent transcriptional increase of all five mucus genes from PND1 to PND6. Furthermore, the relative level of transcription of certain mucus genes on PND1 was increased by the presence of bacteria. This was observed for *Tff3* in the SPF, *Ec,* and *Lb* groups; for *Muc2* in SPF; and for *Muc3* and *Muc4* in *Ec* and *Lb*, respectively.

Detection of bacterial 16S rRNA genes levels above the qPCR detection level occurred only on PND6 and only for some of the colonized animals. On PND6, we found significantly lower levels of *Muc1*, *Muc2* and *Muc4* gene transcription for *Lb* animals with detectable *Lactobacillus* levels as compared to animals with *Lactobacillus* levels below the detection limit.

**Conclusions:**

In summary, our data show that development of the expression of genes encoding secreted (*Muc2*/*Tff3*) and membrane-bound (*Muc1*/*Muc3*/*Muc4*) mucus regulatory proteins, respectively, is distinct and that the onset of this development may be accelerated by specific groups of bacteria present or absent at the mucosal site.

## Background

The interplay between the microbiota of the gut and the intestinal mucus layer in early life is important in the development of the epithelial barrier as part of the innate immune defense [1]. The first weeks and months after birth are believed to be crucial for establishment of the gut microbiota and consequently for the health and integrity of the epithelium throughout life [2,3]. In this period, a development regulated by endogenous factors such as hormones, in parallel with gene regulation caused by the microorganisms present in the gut, takes place [4,5]. The presence and composition of the microbiota has been shown to be directly involved in the regulation of gene transcription in the intestinal epithelium, including the mucin genes, *Muc1-4* and the trefoil factor *Tff3*[4,6].

In the human intestines, MUC1-4 are the most prevalent [6] of the different mucin gene transcripts described to date [1,7,8]. In the gastrointestinal tract, specific mucins show coordinated expression and localization with the viscosity regulating trefoil factors (TFF’s), in particular TFF3 [1]. Epithelial linings contain both membrane-bound (MUC1, MUC3, MUC4) and secreted gel-forming mucins (MUC2) expressing highly specific oligosaccharide side chains, which are important in relation to filtering the entry of various moieties e.g. bacteria and food to the underlying tissue. The membrane-bound mucins act as cell-surface receptors and sensors, mediating signals to trigger cell proliferation, apoptosis, differentiation and specific secretions, when relevant [1]. The four human mucin genes (*MUC1-4*) all share a fairly high degree of sequence, distribution and functional homology to the mouse mucin genes *Muc1-4*[9-12].

As facultative anaerobes, lactobacilli and *E. coli* strains have been recognized as successful early life colonizers of the sterile gastro-intestinal tract [13,14]. Strains of *Lactobacillus acidophilus* are known to stimulate transcription of mucin genes *in vitro*[15,16]. Moreover administrations of probiotic lactobacilli and bifidobacteria have been shown to increase ileal gene and protein levels of *Muc3* in adult rats [17] and cell cultures [16], respectively. Certain *E. coli* strains have been associated with increased production of *MUC2MUC3* and *MUC4* in human ileal cells [18].

In order to elucidate the role of microbial colonization in the postnatal regulation of *Muc1-4* and *Tff3*, we investigated the expression of these genes in mouse ileal segments isolated at the first day after birth (PND1) and six days after birth (PND6), respectively, from specific pathogen free, conventionally bred mice (SPF), mice monocolonized with either *Lactobacillus acidophilus* NCFM (*Lb*) [19] or *E. coli* Nissle (*Ec*) [20,21], and from germ free (GF) mice [15,22]. Specifically, samples were collected and analyzed at PND1 and PND6 to examine the immediate postnatal effects, which are relevant for immune system priming [22,23]. Quantification of bacterial 16S rRNA gene levels was performed to obtain a measure of bacterial colonization levels in the different animal groups on PND1 and PND6.

## Results and discussion

### *qPCR*

We introduced several new primers in this study, all scoring successfully on our validation criteria. LinRegPCR [24,25] was utilized for qPCR analysis, as it enables individual PCR efficiencies to be calculated. The standard curve assumption, that in all samples the PCR efficiency/amplicon, based on one “representative” DNA sample is constant, is replaced by an assumption-free method based on linear regression in the exponential phase of the fluorescence of the actual individual samples analyzed [24]. Further, by including in the subsequent calculation of average efficiency/amplicon, only successful samples within 5 % of the mean efficiency/amplicon, contributions from diverging samples to the final results are excluded.

We tested the choice of reference gene, but interestingly found no significant difference in the results between beta-actin [26,27], neuroplastin (Genevestigator recommendation) nor the geometrical mean of them both.

### Effect of time and bacterial colonization on regulation of Muc1-4 and Tff3 transcription

**Figure 1:**
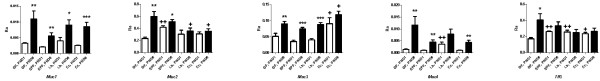
**Comparison of mucin gene expression between the four animal groups on PND1 and PND6.** Transcription of *Muc1*, *Muc2*, *Muc3*, *Muc4* and *Tff3* on postnatal days (PND) 1 and 6 for pups in groups: GF, SPF, *Lb* and *Ec*. Each column represents the average relative abundance (Ra) (See Methods) of 8 animals. Error bars show SEM values. ^*^ p < 0.05, ^**^ p < 0.01, ^***^ p < 0.001 relative to PND1 (for the same group) ^+^ p < 0.05, ^++^ p < 0.01, ^+++^ p < 0.001 relative to GF (for the same day).

In GF mice, *Muc1-4* and *Tff3*, all showed statistically significant increases in transcription from PND1 to PND6, indicating that this event occurs during the first postnatal week independently of the presence of microbes (Figure 1). For certain mucin genes, presence of bacteria in the colonized animals correlated with an increased relative abundance of transcripts on PND1 compared to transcription levels of the same genes in GF mice. This was particularly evident for the genes *Muc2* and *Tff3*. Increased transcription on PND1 of *Tff3* was observed in conventional pups (SPF) as well as in pups of dams’ monocolonized with either *Lb* or *Ec*, while for *Muc2,* this was observed only in presence of a full microbiota (SPF). For *Muc3* in *Ec* and *Muc4* in *Lb*, a higher level of transcription was observed on PND1 than in GF pups, indicating that *E. coli* and *Lactobacillus* may specifically stimulate transcription of these genes immediately after birth (Figure 1).

The higher level of *Muc2* and *Tff3* transcriptions at PND1, both encoding secreted proteins with goblet cell origin [28], indicates that the presence of bacteria affects gene transcription onset in these exocytotic cells. While both gene products play protective roles during gut inflammatory conditions, at sites of epithelial damage [18,29-34] and during postnatal development [35,36], Muc2, unlike Tff3, polymerizes into a protective gel-like structure [1]. Based on the obtained results, it is however not possible to determine whether there is a connection between this difference in functionality and the corresponding gene regulation.

Previously, we demonstrated how microbiota affects ileal gene expression of a number of immune related genes (specific cytokines and chemokines) during the first week after birth [23]. As seen for *Muc2* in the present study, and also for a number of Toll-like receptor signaling pathway related genes such as *Tlr2/4Irak1* and the chemokine *Cxcl2*, encoding MIP-2, the presence of a full microbiota was required to influence gene expression on PND1, which was only to a limited degree affected by monocolonization with either *Lactobacilli* or *E. coli*[23].

Increased transcription of *Muc3* and *Muc4* on PND1 was observed in *Ec* and *Lb* pups, respectively, but not in SPF (Figure 1). Although specific probiotic bacteria, including *Lactobacillus acidophilus* NCFM [15], *Lactobacillus rhamnosus*[17], *Bifidobacterium bifidum*[17], *Lactobacillus plantarum*[16,17] as well as two atypical, enteropathogenic *E.coli* strains [18], have previously been shown to stimulate mucin gene expression, this study is to our knowledge the first to address such effects at a very early stage of life. *Muc1* transcription levels were in this study apparently not affected by the presence of bacteria.

### Bacterial 16S rRNA abundance on PND1 and PND6

None of the PND1 samples contained *Lactobacillus* or *E.coli* in amounts above the qPCR detection limit (DL), in any of the four animal groups (Table 1). This was expected, since only partial bacterial colonization is achieved so short after birth. On PND6, 5/8 pups in both the *Lb* and SPF groups, respectively, were positively above the *Lactobacillus* 16S rRNA DL, while 8/8 and 0/8 in the *Ec* and SPF groups, respectively, were colonized above the *E.coli* DL. These observations corresponded to differences in N_0_ values (See Methods) of >300-fold for *Lactobacillus* and >160-fold for *E.coli*. This shows that bacterial levels in the ileal sections increased between PND1 and PND6 after birth, although the employed procedure did not allow detection of bacterial 16S rRNA in all pups. Culture-based techniques have shown that the gut mucosal surfaces in newborn mice follow a rather conserved colonization pattern [37]. In particular, lactobacilli colonize within the first 1–2 days after birth, whereas coliforms are normally not quantifiable in the mucosal layers until approximately 9 days after birth [14]. These results are thus consistent with findings in the SPF group in this study. It is however important to note, that the current analysis was performed on whole intestinal sections, including both luminal contents and mucosal surfaces, whereas the other studies referred to were based on analysis of mucosal surfaces only.

**Table 1:** 16S rRNA measured presence vs. absence of all 4 animal groups on each of days PND1 and PND6

	**PND1**		**PND6**	
	***Lactobacillus ***	***E. coli ***	***Lactobacillus ***	***E. coli ***
	**16S**	**16S**	**16S**	**16S**
GF	0/8	0/8	0/8	0/8
SPF	0/8	0/8	5/8	0/8
*Ec*	0/8	0/8	0/8	8/8
*Lb*	0/8	0/8	5/8	0/8

The fraction denotes number of samples significantly above detection limit (DL) of the total number (N = 8 in each group).

**Figure 2:**
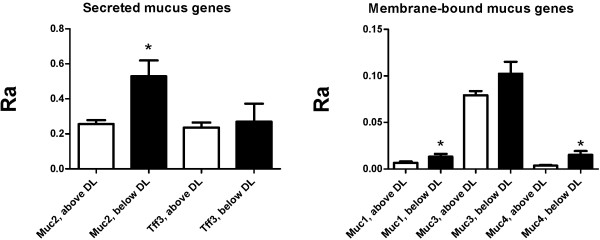
**Difference in mucin gene expression between pups with detectable and undetectable *Lactobacillus *16S in *Lb.*** Comparison of *Muc1*, *Muc2*, *Muc3*, *Muc4* and *Tff3* transcription on PND6 between pups in the *Lb* group, where *Lactobacillus* 16S could be detected and could not be detected, respectively. Each column represents the average relative abundance (Ra) of 5/8 (above DL) or 3/8 (below DL) animals. Error bars show SEM values. DL = Detection Limit. ^*^ p < 0.05, denotes significant difference between detectably colonized and not colonized.

There was a significantly lower level of transcripts (p < 0.05) of *Muc1*, *Muc2* and *Muc4* in the pups with detectable amounts of lactobacilli on PND6 in the *Lb* group than in pups with colonization below the detection limit (Figure 2). In other words, colonization with relatively high levels of *Lactobacilli* in the pups had a negative effect on mucin gene transcription on PND6. For *Muc2*, pups colonized with *Lactobacillus* below the detection limit in the *Lb* group were indeed comparable to GF pups.

It has been established by others that degradation of mucin in adult rats [38] as well as gene expression of *Muc1-4* and *Tff3* in six week old mice [6], is different when comparing GF and conventional animals. Clearly, gene regulation induced by the colonizing microbiota is a complex and continuous process occurring throughout the first weeks of life, and as a more stable and adult-like microbiota is probably not achieved until the end of weaning process at approximately 21 days after birth [39], the expression of the mucus regulating genes may change not only in newborn animals, but also later in life in response to periodic changes in the microbiota

## Conclusions

In this manuscript, we show distinct differences between the expression patterns of secreted (*Muc2*/*Tff3*) and membrane-bound (*Muc1*/*Muc3*/*Muc4*) mucus-regulatory genes in the very first days after birth. Presence of a full microbiota (SPF) increased the relative level of transcription of *Muc2* and *Tff3*, which implies the two corresponding secreted gene products, Muc2 and Tff3, to play protective roles in the postnatal intestinal layer development. The immediate activation of *Muc2*/*Tff3* transcription may provide a coating of the new born ileal epithelial layer, allowing only passage of certain substances or organisms.

## Methods

### Animal experiments

GF Swiss Webster mice and SPF mice, containing conventional microbiota, were purchased from Taconic (Lille Skensved, Denmark) and kept in GF isolators or under specific pathogen-free conditions, respectively [22]. Fecal samples from GF mice, taken at sampling i.e. once a week, were cultivated on non-selective LB medium and under aerobic and anaerobic conditions to confirm sterility of isolators. For breeding, pairs of female mice were housed with one male until plugs were observed. Mono-colonization of pregnant mice with *Ec* and *Lb* was performed 7 days after mating by applying 5x10^8^ CFU ml^-1^ in 0.5 ml PBS suspension orally using a pipette and 0.5 ml to the abdominal skin. *Lb* was grown anaerobically in de Man, Rogosa, and Sharpe broth (MRS, Merck, Darmstadt, Germany) and *Ec* aerobically in Luria-Bertani broth (LB, Merck) overnight at 37°C. The cultures were harvested, washed twice in sterile phosphate-buffered saline (PBS) (Lonza, Basel, Switzerland), re-suspended in 1/50 of the original culture volume and frozen at −80 °C until use. Prior to administering bacteria to the mice, *Ec* suspensions were diluted tenfold in PBS immediately to obtain 5x10^8^ CFU ml^-1^. *Lb* suspensions were not diluted. Four litters spontaneously delivered from 4 different mothers in each group; SPF, GF, *Lb* and *Ec*, were used for the experiment. At post-natal days 1 and 6, the pups were put down and the distal ileum (segment from cecum and 3 cm up) was removed from the small intestine of two pups per litter and frozen in RNAlater (Qiagen, Hilden, Germany). No separation of mucosal from luminal content was performed.

### Ethics

The mouse experiment was performed under a license to Department of Microbiology, National Food Institute, from the Danish Council for Animal Experimentation.

### RNA isolation

Tissues were removed from RNAlater and homogenized by a rotor strator in RLT buffer (Qiagen). RNA from tissue homogenate was extracted using RNeasy Mini Kit from Qiagen following the supplier's protocol.

### Primer design and validation

A list of all primers used in this study is presented in Table 2. All primers found in references were initially checked with the Net Primer Software (http://www.premierbiosoft.com/netprimer/index.html). Primers not scoring a rating of at least 90 % were not accepted and new primers were then designed with NCBI’s primer designing tool (http://www.ncbi.nlm.nih.gov/tools/primer-blast/) and the quality was again verified until satisfaction with the Net Primer Software. All newly designed primers were designed to span exon junctions to avoid amplification of genomic DNA. The specificity of all primers was evaluated *in silico* using nucleotide BLAST, (http://blast.ncbi.nlm.nih.gov/Blast.cgi).

**Table 2:** Primers used for qPCR

**Primer name**	**Fwd (5´-3´)**	**Rev (5´-3´)**	**Amplicon size**	**Reference**
*Muc1*	TCGTCTATTTCCTTGCCCTG	ATTACCTGCCGAAACCTCCT	185	This study
*Muc2*	CCCAGAAGGGACTGTGTATG	TTGTGTTCGCTCTTGGTCAG	276	Modified from [44]
*Muc3*	TGGTCAACTGCGAGAATGGA	TACGCTCTCCACCAGTTCCT	98	Modified from [6]
*Muc4*	GTCTCCCATCACGGTTCAGT	TGTCATTCCACACTCCCAGA	280	This study
*Tff3*	CTCTGTCACATCGGAGCAGTGT	TGAAGCACCAGGGCACATT	77	[45]
*Neuroplastin*	CGCTGCTCAGAACGAACCAAGAA	CTTACGGGTGGCAGTGAGTT	160	Modified from [46]
*Beta-actin*	GTCCACCTTCCAGCAGATGT	GAAAGGGTGTAAAACGCAGC	117	This study
*Lactobacillus* 16S rRNA	AGCAGTAGGGAATCTTCCA^a^	CACCGCTACACATGGAG^b^	341	^a^[47] ^b^[48]
*E. coli* 16S rRNA	CATGCCGCGTGTATGAAGAA	CGGGTAACGTCAATGAGCAAA	96	[49]

### Quantitative PCR (qPCR)

Isolated ileal RNA was reverse transcribed into cDNA using SuperScript® VILO™ cDNA Synthesis Kit from Invitrogen, Denmark. After verifying the quality of the cDNA by spectroscopy (A_260_/A_280_ = 1.8 ± 10 %) measured on a NanoDrop ND-1000 Spectrophotometer (Saveen Werner, Limhamn, Sweden), it was used as template in quantitative real-time PCR using the ABI prism 7900HT from Applied Biosystems. All cDNA concentrations were within the range of 90-100 ng μl^-1^. The amplification reactions were carried out in a total volume of 20 μl containing 10 μl master mix (2x PerfeCTa^TM^ SYBR® Green SuperMix, ROX from Quanta Biosciences ^TM^), 0.4 μl of each primer (10 μM), 2 μl template cDNA, and 7.2 μl nuclease-free water (Qiagen GmbH, Germany) purified for PCR. The amplification program consisted of one cycle of 50 °C for 2 min; one cycle of 95 °C for 10 min; 40 cycles of 95 °C for 15 s and 60 °C for 1 min; and finally one cycle of dissociation curve analysis for assessing the amplification products (95 °C for 15 s, 60 °C for 20s and increasing ramp rate by 2 % until 95 °C for 15 s). These conditions were selected based on preliminary qPCR experiments on target DNA with similar concentrations (100 ng μl^-1^). Samples of all amplification products were further subjected to gel electrophoresis in 2 % agarose, followed by ethidium bromide staining in order to verify amplicon sizes.

### qPCR setup

Three separate qPCR experiments on ileal cDNA were performed; 1) and 2) were separate replications of relative quantifications on mucus gene transcription (*Muc1-4* and *Tff3*) with selected reference genes (see next paragraph) and 3) on presence or absence of specific bacterial 16S rRNA analysis (*Lactobacillus*, *E.coli*).

### qPCR data analysis

All qPCR analysis was performed with the freely available LinRegPCR tool developed by Ruijter et al. [24,25]. The raw fluorescence data were exported from the ABI prism 7900HT SDS-software, and the LinRegPCR program was used to estimate baselines and individual PCR efficiencies in order to calculate output as target starting concentration, expressed in arbitrary fluorescence units N_0_, for each PCR sample by the formula N_0_ = threshold / (Eff_mean_^Ct^), where Eff_mean_ denotes the optimal PCR mean efficiency/amplicon, threshold the optimal “cutoff” in the exponential region and C_t_ is the number, where each PCR sample exceeds this threshold. Samples with no amplification, baseline error, too much noise or without plateau were automatically excluded by the LinRegPCR software. Subsequently, for each amplicon the average of all remaining, successful samples within 5 % of the mean value of all successful samples/amplicon were used in the calculation of Eff_mean_ for each amplicon. All N_0_-values were calculated as means of double qPCR determinations.

For relative quantification of mucus gene transcripts, two different eukaryotic reference genes were used namely beta-actin [40] and neuroplastin, the latter suggested by the Genevestigator software (https://www.genevestigator.com) [41,42] based on microarray data on similar organism (*M. musculus*) and tissue (ileum). We used the geometric mean of the two reference genes as previously suggested [43]. Normalization to relevant reference gene expression was then calculated according to the formula: Ra =Ratio = N_0_^Sample^/ N_0_^Reference^ and averaged over the two qPCR experiments.

Unspecific amplification of 16S rRNA bacterial genes from GF mice was used to specify detection limits for specific amplifications (*Lactobacillus*, *E .coli*). Cutoffs for presence of either bacterium were defined by at least 5 C_t_-values difference from the GF samples. No normalization to reference genes and thus relative quantification was used for the 16S analysis, since the purpose was only to determine presence vs. absence of detectable bacteria.

### Statistics

All statistics was performed with GraphPad Prism 5. One-way ANOVA followed by Dunnett’s *post hoc* test with GF as control group and Student’s *t*-test was used to compare mucus gene expression between the four animal groups and development from PND1 to PND6, respectively. P-values lower than p = 0.05 were considered statistically significant. Welch’s correction for unequal variances was applied, when necessary.

## Competing interests

The authors declare that they have no competing interests.

## Authors’ contributions

AB performed the qPCR experiments, including cDNA syntheses, data interpretation and statistical analysis, and wrote the manuscript. MBK and SBM performed the animal experiments, including isolation of ileal tissue and RNA purification. TRL, HF and LNF conceived of the study setup and participated in its design and coordination. TRL, MBK, HF and MIB contributed to data analysis and interpretation as well as preparation of the manuscript. All authors read and approved the final manuscript.

## References

[B1] Hollingsworth MA Swanson BJ Mucins in cancer: protection and control of the cell surface Nat Rev Cancer 2004 4 45 60 10.1038/nrc1251 14681689

[B2] Adlerberth I Wold AE Establishment of the gut microbiota in Western infants Acta Paediatr 2009 98 229 238 10.1111/j.1651-2227.2008.01060.x 19143664

[B3] Fanaro S Chierici R Guerrini P Vigi V Intestinal microflora in early infancy: composition and development Acta Paediatr Suppl 2003 91 48 55 14599042 10.1111/j.1651-2227.2003.tb00646.x

[B4] Hooper LV Wong MH Thelin A Hansson L Falk PG Gordon JI Molecular analysis of commensal host-microbial relationships in the intestine Science 2001 291 881 884 10.1126/science.291.5505.881 11157169

[B5] Favier CF Vaughan EE de Vos WM Akkermans AD Molecular monitoring of succession of bacterial communities in human neonates Appl Environ Microbiol 2002 68 219 226 10.1128/AEM.68.1.219-226.2002 11772630 PMC126580

[B6] Comelli EM Simmering R Faure M Donnicola D Mansourian R Rochat F Corthesy-Theulaz I Cherbut C Multifaceted transcriptional regulation of the murine intestinal mucus layer by endogenous microbiota Genomics 2008 91 70 77 10.1016/j.ygeno.2007.09.006 18035521

[B7] Corfield AP Myerscough N Longman R Sylvester P Arul S Pignatelli M Mucins and mucosal protection in the gastrointestinal tract: new prospects for mucins in the pathology of gastrointestinal disease Gut 2000 47 589 594 10.1136/gut.47.4.589 10986224 PMC1728059

[B8] McGuckin MA Linden SK Sutton P Florin TH Mucin dynamics and enteric pathogens Nat Rev Microbiol 2011 9 265 278 10.1038/nrmicro2538 21407243

[B9] Xing PX Lees C Lodding J Prenzoska J Poulos G Sandrin M Gendler S McKenzie IF Mouse mucin 1 (MUC1) defined by monoclonal antibodies Int J Cancer 1998 76 875 883 10.1002/(SICI)1097-0215(19980610)76:6<875::AID-IJC18>3.0.CO;2-1 9626356

[B10] Shekels LL Hunninghake DA Tisdale AS Gipson IK Kieliszewski M Kozak CA Ho SB Cloning and characterization of mouse intestinal MUC3 mucin: 3' sequence contains epidermal-growth-factor-like domains Biochem J 1998 330 Pt 3 1301 1308 9494100 10.1042/bj3301301 PMC1219276

[B11] van Klinken BJ Einerhand AW Duits LA Makkink MK Tytgat KM Renes IB Verburg M Büller HA Gastrointestinal expression and partial cDNA cloning of murine Muc2 Am J Physiol 1999 276 G115 G124 9886986 10.1152/ajpgi.1999.276.1.G115

[B12] Desseyn JL Clavereau I Laine A Cloning, chromosomal localization and characterization of the murine mucin gene orthologous to human MUC4 Eur J Biochem 2002 269 3150 3159 10.1046/j.1432-1033.2002.02988.x 12084055

[B13] Salminen S Isolauri E Intestinal colonization, microbiota, and probiotics J Pediatr 2006 149 S115 S120 10.1016/j.jpeds.2006.06.062

[B14] Savage DC Dubos R Schaedler RW The gastrointestinal epithelium and its autochthonous bacterial flora J Exp Med 1968 127 67 76 10.1084/jem.127.1.67 4169441 PMC2138434

[B15] Sanders ME Klaenhammer TR Invited review: the scientific basis of Lactobacillus acidophilus NCFM functionality as a probiotic J Dairy Sci 2001 84 319 331 10.3168/jds.S0022-0302(01)74481-5 11233016

[B16] Mack DR Ahrne S Hyde L Wei S Hollingsworth MA Extracellular MUC3 mucin secretion follows adherence of Lactobacillus strains to intestinal epithelial cells in vitro Gut 2003 52 827 833 10.1136/gut.52.6.827 12740338 PMC1773687

[B17] Dykstra NS Hyde L Adawi D Kulik D Ahrne S Molin G Jeppsson B Mackenzie A Mack DR Pulse probiotic administration induces repeated small intestinal Muc3 expression in rats Pediatr Res 2011 69 206 211 10.1203/PDR.0b013e3182096ff0 21135754

[B18] Vieira MA Gomes TA Ferreira AJ Knobl T Servin AL Lievin-Le M V: Two atypical enteropathogenic Escherichia coli strains induce the production of secreted and membrane-bound mucins to benefit their own growth at the apical surface of human mucin-secreting intestinal HT29-MTX cells Infect Immun 2010 78 927 938 10.1128/IAI.01115-09 20065027 PMC2825950

[B19] Wright CT Klaenhammer TR Calcium-induced alteration of cellular morphology affecting the resistance of lactobacillus acidophilus to freezing Appl Environ Microbiol 1981 41 807 815 16345739 10.1128/aem.41.3.807-815.1981 PMC243778

[B20] Nissle A Mutaflor and its medical significance Z Klin Med 1951 2 68 14800874

[B21] Jacobi CA Malfertheiner P Escherichia coli Nissle 1917 (Mutaflor): new insights into an old probiotic bacterium Dig Dis 2011 29 600 607 10.1159/000333307 22179217

[B22] Zeuthen LH Fink LN Metzdorff SB Kristensen MB Licht TR Nellemann C Frøkiær H Lactobacillus acidophilus induces a slow but more sustained chemokine and cytokine response in naive foetal enterocytes compared to commensal Escherichia coli BMC Immunol 2010 11 2 10.1186/1471-2172-11-2 20085657 PMC2831831

[B23] Fink LN Metzdorff SB Zeuthen LH Nellemann C Kristensen MB Licht TR Frøkiær H Establishment of tolerance to commensal bacteria requires a complex microbiota and is accompanied by decreased intestinal chemokine expression Am J Physiol Gastrointest Liver Physiol 2012 302 1 G55 G65 10.1152/ajpgi.00428.2010 21960522

[B24] Ramakers C Ruijter JM Deprez RH Moorman AF Assumption-free analysis of quantitative real-time polymerase chain reaction (PCR) data Neurosci Lett 2003 339 62 66 10.1016/S0304-3940(02)01423-4 12618301

[B25] Ruijter JM Ramakers C Hoogaars WM Karlen Y Bakker O van den Hoff MJ Moorman AF Amplification efficiency: linking baseline and bias in the analysis of quantitative PCR data Nucleic Acids Res 2009 37 e45 10.1093/nar/gkp045 19237396 PMC2665230

[B26] Bustin SA Absolute quantification of mRNA using real-time reverse transcription polymerase chain reaction assays J Mol Endocrinol 2000 25 169 193 10.1677/jme.0.0250169 11013345

[B27] Veazey KJ Golding MC Selection of stable reference genes for quantitative rt-PCR comparisons of mouse embryonic and extra-embryonic stem cells PLoS One 2011 6 e27592 10.1371/journal.pone.0027592 22102912 PMC3213153

[B28] Bergstrom KS Guttman JA Rumi M Ma C Bouzari S Khan MA Gibson DL Vogl AW Vallance BA Modulation of intestinal goblet cell function during infection by an attaching and effacing bacterial pathogen Infect Immun 2008 76 796 811 10.1128/IAI.00093-07 17984203 PMC2223480

[B29] Dignass AU Sturm A Peptide growth factors in the intestine Eur J Gastroenterol Hepatol 2001 13 763 770 10.1097/00042737-200107000-00002 11474304

[B30] Heazlewood CK Cook MC Eri R Price GR Tauro SB Taupin D Thornton DJ Png CW Crockford TL Cornall RJ Adams R Kato M Nelms KA Hong NA Florin TH Goodnow CC McGuckin MA Aberrant mucin assembly in mice causes endoplasmic reticulum stress and spontaneous inflammation resembling ulcerative colitis PLoS Med 2008 5 e54 10.1371/journal.pmed.0050054 18318598 PMC2270292

[B31] Boshuizen JA Reimerink JH Korteland-Van Male AM Van HV Bouma J Gerwig GJ Koopmans MP Büller HA Dekker J Einerhand AW Homeostasis and function of goblet cells during rotavirus infection in mice Virology 2005 337 210 221 10.1016/j.virol.2005.03.039 15882887

[B32] Taupin D Podolsky DK Trefoil factors: initiators of mucosal healing Nat Rev Mol Cell Biol 2003 4 721 732 14506475 10.1038/nrm1203

[B33] Tran CP Cook GA Yeomans ND Thim L Giraud AS Trefoil peptide TFF2 (spasmolytic polypeptide) potently accelerates healing and reduces inflammation in a rat model of colitis Gut 1999 44 636 642 10.1136/gut.44.5.636 10205199 PMC1727500

[B34] Thim L Madsen F Poulsen SS Effect of trefoil factors on the viscoelastic properties of mucus gels Eur J Clin Invest 2002 32 519 527 10.1046/j.1365-2362.2002.01014.x 12153553

[B35] Scholven J Taras D Sharbati S Schon J Gabler C Huber O Meyer Zum Büschenfelde D Blin N Einspainer R Intestinal expression of TFF and related genes during postnatal development in a piglet probiotic trial Cell Physiol Biochem 2009 23 143 156 10.1159/000204103 19255509

[B36] Fanca-Berthon P Michel C Pagniez A Rival M Van SI Darmaun D Hoebler C Intrauterine growth restriction alters postnatal colonic barrier maturation in rats Pediatr Res 2009 66 47 52 10.1203/PDR.0b013e3181a2047e 19287349

[B37] Schaedler RW Dubos R Costello R The development of the bacterial flora in the gastrointestinal tract of mice J Exp Med 1965 122 59 66 10.1084/jem.122.1.59 14325473 PMC2138024

[B38] Midtvedt T Carlstedt-Duke B Hoverstad T Midtvedt AC Norin KE Saxerholt H Establishment of a biochemically active intestinal ecosystem in ex-germfree rats Appl Environ Microbiol 1987 53 2866 2871 3124742 10.1128/aem.53.12.2866-2871.1987 PMC204214

[B39] Davis CP McAllister JS Savage DC Microbial colonization of the intestinal epithelium in suckling mice Infect Immun 1973 7 666 672 4586864 10.1128/iai.7.4.666-672.1973 PMC422740

[B40] Huggett J Dheda K Bustin S Zumla A Real-time RT-PCR normalisation; strategies and considerations Genes Immun 2005 6 279 284 10.1038/sj.gene.6364190 15815687

[B41] Laule O Hirsch-Hoffmann M Hruz T Gruissem W Zimmermann P Web-based analysis of the mouse transcriptome using Genevestigator BMC Bioinformatics 2006 7 311 10.1186/1471-2105-7-311 16790046 PMC1533866

[B42] Zimmermann P Hirsch-Hoffmann M Hennig L Gruissem W Genevestigator. Arabidopsis microarray database and analysis toolbox Plant Physiol 2004 136 2621 2632 10.1104/pp.104.046367 15375207 PMC523327

[B43] Vandesompele J De PK Pattyn F Poppe B Van RN De Paepe A Speleman F Accurate normalization of real-time quantitative RT-PCR data by geometric averaging of multiple internal control genes Genome Biol 2002 3 7 research0034.1-0034.11 12184808 10.1186/gb-2002-3-7-research0034 PMC126239

[B44] Tai EK Wu WK Wong HP Lam EK Yu L Cho CH A new role for cathelicidin in ulcerative colitis in mice Exp Biol Med (Maywood) 2007 232 799 808 17526772

[B45] Liu J Yu L Tokar EJ Bortner C Sifre MI Sun Y Waalkes MP Arsenic-induced aberrant gene expression in fetal mouse primary liver-cell cultures Ann N Y Acad Sci 2008 1140 368 375 10.1196/annals.1454.028 18991936 PMC2697955

[B46] Kreutz MR Langnaese K Dieterich DC Seidenbecher CI Zuschratter W Beesley PW Gundelfinger ED Distribution of transcript and protein isoforms of the synaptic glycoprotein neuroplastin in rat retina Invest Ophthalmol Vis Sci 2001 42 1907 1914 11431460

[B47] Walter J Hertel C Tannock GW Lis CM Munro K Hammes WP Detection of Lactobacillus, Pediococcus, Leuconostoc, and Weissella species in human feces by using group-specific PCR primers and denaturing gradient gel electrophoresis Appl Environ Microbiol 2001 67 2578 2585 10.1128/AEM.67.6.2578-2585.2001 11375166 PMC92910

[B48] Heilig HG Zoetendal EG Vaughan EE Marteau P Akkermans AD De Vos WM Molecular diversity of Lactobacillus spp. and other lactic acid bacteria in the human intestine as determined by specific amplification of 16S ribosomal DNA Appl Environ Microbiol 2002 68 114 123 10.1128/AEM.68.1.114-123.2002 11772617 PMC126540

[B49] Huijsdens XW Linskens RK Mak M Meuwissen SG Vandenbroucke-Grauls CM Savelkoul PH Quantification of bacteria adherent to gastrointestinal mucosa by real-time PCR J Clin Microbiol 2002 40 4423 4427 10.1128/JCM.40.12.4423-4427.2002 12454130 PMC154607

